# Apatorsen plus docetaxel versus docetaxel alone in platinum-resistant metastatic urothelial carcinoma (Borealis-2)

**DOI:** 10.1038/s41416-018-0087-9

**Published:** 2018-05-16

**Authors:** Jonathan E. Rosenberg, Noah M. Hahn, Meredith M. Regan, Lillian Werner, Ajjai Alva, Saby George, Joel Picus, Robert Alter, Arjun Balar, Jean Hoffman-Censits, Petros Grivas, Richard Lauer, Elizabeth A. Guancial, Christopher Hoimes, Guru Sonpavde, Constantine Albany, Mark N. Stein, Tim Breen, Cindy Jacobs, Kirsten Anderson, Joaquim Bellmunt, Aly-Khan A. Lalani, Sumanta Pal, Toni K. Choueiri

**Affiliations:** 1 Memorial Sloan Kettering Cancer Center, New York, NY USA; 2 Sidney Kimmel Comprehensive Cancer Center, Johns Hopkins University, Baltimore, MD USA; 3 Dana-Farber Cancer Institute, Boston, MA USA; 4 University of Michigan Comprehensive Cancer Center, Ann Arbor, MI USA; 5 Roswell Park Cancer Institute, Buffalo, NY USA; 6 Siteman Cancer Center, Washington University, St. Louis, MO USA; 7 John Theurer Cancer Center, Hackensack University Medical Center, Hackensack, NJ USA; 8 New York University Perlmutter Cancer Center, New York, NY USA; 9 Sidney Kimmel Cancer Center at Jefferson, Philadelphia, PA USA; 10 Cleveland Clinic Taussig Cancer Institute, Cleveland, OH USA; 11 University of New Mexico Cancer Center, Albuquerque, NM USA; 12 University of Rochester Wilmot Cancer Institute, Rochester, NY USA; 13 University Hospitals Seidman Cancer Center, Cleveland, OH USA; 14 University of Alabama Comprehensive Cancer Center, Birmingham, AL USA; 15 Indiana University Melvin and Bren Simon Cancer Center, Indianapolis, IN USA; 16 Rutgers Cancer Institute of New Jersey, New Brunswick, NJ USA; 17 Hoosier Cancer Research Network, Indianapolis, IN USA; 18 OncoGenex Pharmaceuticals, Inc., Bothell, WA USA; 19 City of Hope Comprehensive Cancer Center, Duarte, CA USA

**Keywords:** Bladder cancer, Prognostic markers

## Abstract

**Background:**

A randomised study to assess the addition of apatorsen, an antisense oligonucleotide that inhibits Hsp27 expression, to docetaxel in patients with metastatic urothelial carcinoma (mUC) relapsed after prior platinum-based chemotherapy.

**Methods:**

Multicentre, phase II study with 1:1 randomisation to apatorsen (three loading doses at 600 mg intravenous followed by weekly doses) plus docetaxel (75 mg/m^2^ intravenous every 21 days) (A/D) or docetaxel alone. Overall survival (OS) was the primary end point with a *P* value <0.1 (one-sided) being positive. Progression-free survival (PFS), objective response rate (ORR), safety, and effect of Hsp27 levels on outcomes were secondary end points.

**Results:**

Patients randomised to A/D (*n* = 99) had improved OS compared to docetaxel alone (*n* = 101): HR: 0.80, 80% CI: 0.65–0.98, *P* = 0.0784, median 6.4 vs 5.9 months. PFS and ORR were similar in both arms. A/D had more incidence of sepsis and urinary tract infections. Patients with baseline Hsp27 levels <5.7 ng/mL had improved OS compared to those with levels ≥5.7 ng/mL. Patients with a decline or ≤20.5% increase in Hsp27 from baseline benefited more from A/D than those with >20.5% increase.

**Conclusions:**

A/D met its predefined OS end point in patients with platinum-refractory mUC in this phase II trial. This trial is hypothesis generating requiring further study before informing practice.

## Introduction

Heat shock proteins (Hsp) are a family of highly conserved proteins whose expression is induced by cell stressors such as hyperthermia, oxidative stress, cytotoxic chemotherapy, and radiation.^1^ Hsp27 is highly expressed in many cancers and is associated with poor prognosis.^1,2^ Hsp27 also stabilises mutated or inappropriately activated oncoproteins that contribute to the initiation, growth, and metastasis of human cancers.^3–7^ While Hsp27 is expressed in low levels in normal bladder epithelium,^8^ expression is increased in bladder cancer.^9–11^

Apatorsen (OGX-427) is an antisense oligonucleotide (ASO) designed to bind to Hsp27 mRNA, resulting in the inhibition of the production of Hsp27 protein.^12,13^ Apatorsen is similar to endogenous DNA but contains second-generation ASO chemical modifications intended to optimise its pharmacological potency, pharmacokinetics, and safety profile. In vitro and in vivo evidence indicates that Hsp27 inhibition leads to inhibition of tumour growth and sensitisation to cytotoxic chemotherapy,^14,15^ and a trial of apatorsen as intravesical therapy for non-muscle invasive bladder cancer showed promising anticancer activity.^16^ Phase I studies of apatorsen as a single agent and in combination with docetaxel appeared to be well tolerated even at the highest dose of 1000 mg.^17^

We report the efficacy and safety of apatorsen in combination with docetaxel compared to docetaxel alone in patients with metastatic urothelial carcinoma previously treated with platinum-based chemotherapy. This randomised, controlled phase II trial with a primary end point of overall survival was designed to provide a strong rationale for whether to move forward with a phase III trial in this patient population.

## Patients and methods

### Study design and participants

This was a randomised, phase II, investigator-sponsored, multicentre, open-label trial conducted among academic and community sites within the Hoosier Cancer Research Network (HCRN). Patients with metastatic or locally advanced inoperable urothelial carcinoma (TNM staging T4b, N2, N3, or M1) previously treated with platinum-based chemotherapy were enrolled. To be eligible, patients 18 years or older were required to have measurable disease, an Eastern Cooperative Oncology Group (ECOG) performance status of 0 or 1, and estimated life expectancy of 3 or more months. All patients must have received at least one prior platinum-based chemotherapy regimen with a maximum of two regimens. Patients who relapsed within 1 year of platinum-based perioperative chemotherapy were eligible. Patients whose tumours contained variant histological features were eligible if the tumour was not considered a pure histologic variant; however, patients with any amount of small cell carcinoma were not eligible. Patients were required to have adequate organ function (serum creatinine ≤1.5× upper limit of normal (ULN)), no worse than grade 1 peripheral neuropathy, no known brain or spinal cord metastases, no active second malignancy, no cerebrovascular accident, myocardial infarction, or pulmonary embolus within 3 months of enrollment, and no prior treatment with docetaxel.

### Randomisation

Patients were randomly assigned to apatorsen plus docetaxel or docetaxel alone in a one-to-one ratio using permuted blocks within strata. Randomisation was generated using a clinical trial management system software (OnCore) and patients were stratified based on 0 vs 1–3 adverse Bellmunt prognostic factors (liver metastases, haemoglobin <10 g/dL, ECOG performance status 1) and time from prior systemic chemotherapy (<3 months vs ≥3 months).^18,19^

### Procedures

For patients assigned apatorsen plus docetaxel, apatorsen 600 mg was administered intravenously in three separate loading doses separated by at least one non-treatment day over a 9-day period. Patients were administered an antihistamine or an H2 antagonist prior to each of the three loading doses. Following the loading doses, patients received docetaxel 75 mg/m^2^ in 21-day cycles and apatorsen 600 mg weekly until disease progression, unacceptable toxicity, or a maximum of 10 cycles of docetaxel. Patients who completed 10 cycles or stopped docetaxel for toxicity continued maintenance apatorsen until disease progression or unacceptable toxicity related to apatorsen. For patients assigned docetaxel alone, docetaxel was administered at a dose of 75 mg/m^2^ every 21 days until disease progression, unacceptable toxicity, or a maximum of 10 cycles of docetaxel. Dose reductions for docetaxel (from 75 mg/m^2^ to 60 mg/m^2^ to 45 mg/m^2^) were required for haematologic toxicity, peripheral sensory neuropathy, or mucositis. Dose reductions for apatorsen (from 600 mg to 500 mg to 400 mg) were required for renal toxicity, and dose reductions for both agents were required for hepatotoxicity.

### Study end points

The primary end point was overall survival (OS), defined from randomisation until death due to any cause, or censored on date last known alive. Secondary efficacy end points were progression-free survival (PFS), defined from randomisation to objective disease progression or death from any cause, whichever occurred first, or censored at date of last disease evaluation without progression; objective response rate (ORR; complete or partial response as best overall response) and duration of response, which were evaluated by RECIST criteria version 1.1. Radiographic evaluations were performed at baseline with cross-sectional imaging and repeated every 6 weeks until disease progression. Patients with bone metastases on baseline bone scan were required to have imaging every 6 weeks for the first 4 cycles and then every 12 weeks thereafter until disease progression. If any new clinical signs or symptoms of disease progression developed, imaging was repeated as clinically indicated. Safety was reported according to National Cancer Institute (NCI) Common Terminology Criteria for Adverse Events (CTCAE version 4.0) and assessed from initiation of study treatment until 30 days after last study therapy. Exploratory objectives included assessing the associations of baseline and post-treatment serum Hsp27 levels with survival outcomes. Hsp27 levels were analysed by a central laboratory using enzyme-linked immunosorbent assay analysis, as has been previously described.^20^

### Statistical analysis

This phase II study was designed to have 90% power with one-sided 0.10 significance level to detect a 33% reduction in the OS hazard rate with docetaxel and apatorsen compared with docetaxel alone [hazard ratio (docetaxel and apatorsen/docetaxel) = 0.667], assuming an exponential distribution of OS, and median OS of 6 months on docetaxel alone.^21^ The specified phase II error levels were considered to provide adequate precision of the hazard ratios (HR) in order to inform the design of a subsequent phase III trial. The randomised, controlled design specified one interim analysis for futility after ~81 deaths and final analysis after 162 deaths. The cutoff date for final analysis was 10 October 2016.

Patient and clinical characteristics were summarised as numbers and percentage for categorical variables and median with range for continuous variables. OS and PFS were compared between the two treatment assignments using a stratified log-rank test with a one-sided *α* = 0.10. The Kaplan–Meier (KM) method was used to estimate OS and PFS distributions by treatment arm. Stratified Cox proportional hazards (PH) models estimated hazard ratios (HR) and 80% two-sided confidence intervals, which corresponds to one-sided *α* = 0.10, in unadjusted and multivariable models. Subgroup analyses investigated treatment effects according to the stratification factors, estimating HRs and testing for treatment-by-subgroup interaction in Cox PH models.

ORR was summarised as numbers and percentage of participants by treatment assignments with two-sided 80% CI and compared using Fisher’s exact tests. Median duration of response was estimated using KM method in patients who achieved partial or complete response as best overall response, defined from time objective response was first observed until disease progression or death. A planned stratified Cox PH model assessed the association of baseline serum Hsp27 level with OS and to test the treatment-by-Hsp27 interaction. Baseline serum Hsp27 levels were categorized at the median for assessing the associations with OS, given that no clinically meaningful cutoff point had been previously established. Among patients who were alive after cycle 2, the association of percentage change of Hsp27 level from baseline to end of cycle 2 with OS (re-defined from end of cycle 2 as landmark analysis) was investigated similarly.

## Results

### Patients

**Fig. 1:**
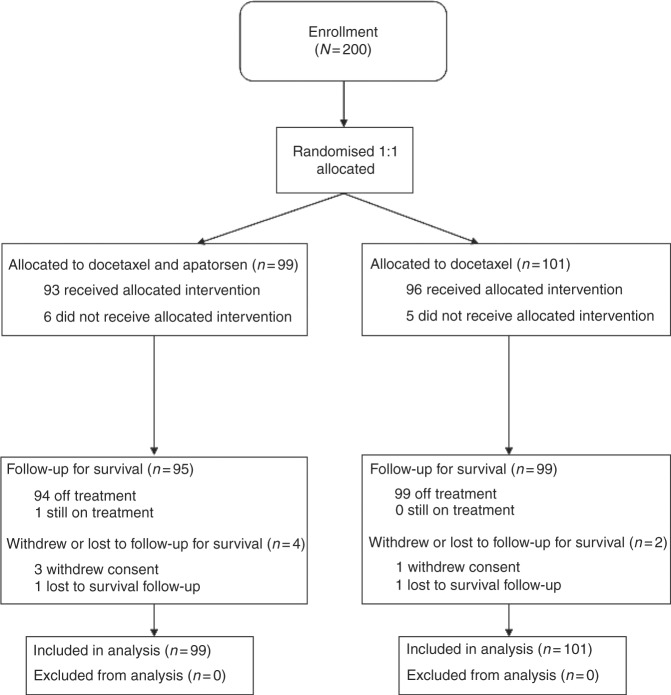
Trial CONSORT flow diagram

Between August 2013 and September 2015, 200 patients were enrolled at 32 study sites in the United States. Ninety-nine patients were randomised to docetaxel and apatorsen and 101 patients to treatment with docetaxel alone. Overall, 194 participants had complete follow-up for survival and 6 were lost to follow-up or withdrew consent without survival follow-up. All 200 participants were included in the intention-to-treat (ITT) analysis population (Fig. 1). Baseline characteristics were well balanced as shown in Table 1. Overall, median age of participants was 67 years (interquartile range, IQR 59–74) and 149 participants (74.5%) were male. Eighty-four (42.0%) had ECOG performance status of 0 at screening. In terms of patient stratification, 140 (70%) had 1–3 Bellmunt prognostic factors and 87 (43.5%) had time from prior systemic chemotherapy <3 months.

**Table 1:** Baseline characteristics

	Treatment assignment	
Characteristic, *n* (%)	Apatorsen and docetaxel (*n* = 99)	Docetaxel (*n* = 101)
Age, median years (range)	68 (43–90)	67 (35–92)
Sex		
Male	74 (74.7%)	75 (74.3%)
Female	25 (25.3%)	26 (25.6%)
Race		
Caucasian	89 (89.9%)	92 (91.1%)
African American	3 (3%)	4 (4%)
Asian	5 (5.1%)	3 (3%)
Unknown	2 (2%)	2 (2%)
ECOG performance status^a^		
0	43 (43.4%)	41 (40.6%)
1	56 (56.6%)	59 (58.4%)
Urothelial carcinoma (at study entry)		
Metastatic	85 (85.9%)	87 (86.1%)
Locally advanced	6 (6.1%)	10 (9.9%)
Unknown	8 (8.1%)	4 (4%)
Primary surgery		
Yes	40 (40.4%)	36 (35.6%)
Prior cisplatin use		
Yes	70 (70.7%)	72 (71.3%)
Prior carboplatin use		
Yes	38 (38.4%)	41 (40.6%)
Primary disease site(s)		
Bladder	64 (64.6%)	72 (71.3%)
Renal pelvis	27 (27.3%)	13 (12.9%)
Ureter	13 (13.1%)	14 (13.9%)
Urethra	7 (7.1%)	9 (8.9%)
Metastatic sites		
Liver	28 (28.3%)	25 (24.8%)
Lung	34 (34.3%)	35 (34.7%)
Bone	19 (19.2%)	21 (20.8%)
Lymph nodes	56 (56.6%)	52 (51.5%)
Bellmunt prognostic factors^b^		
0	27 (27.3%)	32 (31.7%)
1	42 (42.4%)	35 (34.7%)
2	23 (23.2%)	26 (25.7%)
3	7 (7.1%)	7 (6.9%)

Prior paclitaxel use was balanced between both arms (*n* = 10 arm A and *n* = 12 arm B).

*ECOG* Eastern Cooperative Oncology Group.

^a^One patient in the docetaxel arm had ECOG performance status of 2.

^b^One patient in the docetaxel arm had unknown Bellmunt prognostic factors

### Therapy administration

Among patients who started docetaxel, a median of two cycles were received in both groups and among those who received apatorsen, a median of 6 weeks (or doses) of treatment were received. Seven patients went on to receive maintenance apatorsen after stopping docetaxel, with additional weeks of apatorsen reported as: 1, 2, 3, 7, 7, 33, and 61 weeks for these patients.

### Primary end point: overall survival

**Fig. 2:**
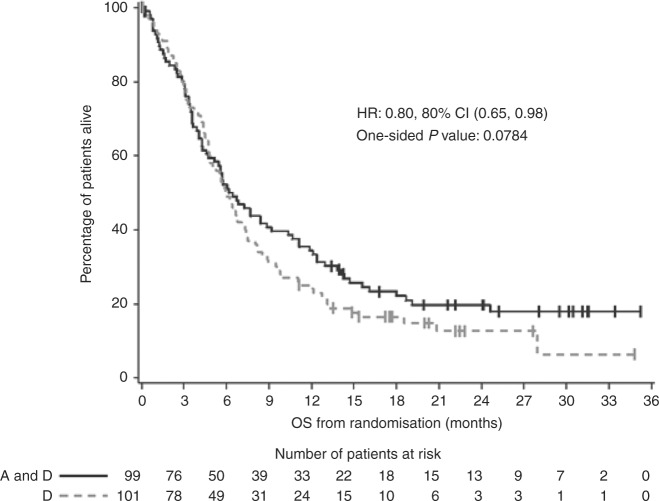
Kaplan–Meier estimate of overall survival (OS). A apatorsen, D docetaxel, HR hazard ratio, CI confidence interval

Median follow-up time for all surviving patients was 21.6 months (range, <1–35.3 months). At the time of analysis, 163 deaths were reported, with 77 (77.8%) assigned docetaxel and apatorsen and 86 (85.1%) assigned docetaxel. Patients assigned to docetaxel and apatorsen had a reduction in hazard of death as compared to patients assigned docetaxel alone (HR: 0.80; 80% CI: 0.65–0.98, one-sided *P* = 0.0784, median OS 6.4 vs 5.9 months). The estimated 12-month OS was 34.4% and 25.0% among patients assigned to docetaxel and apatorsen vs docetaxel alone, respectively (Fig. 2).

### Secondary end points and subgroup analysis

Patients assigned to docetaxel and apatorsen had a reduced hazard of disease progression or death as compared to patients assigned to docetaxel alone, although the results were not statistically significant (HR: 0.80, 80% CI: 0.64–1.01, one-sided *P* = 0.1069, median PFS 1.8 vs 1.6 months, estimated 12-month PFS 8.7% vs 3.8%).

One-hundred and forty-five patients had evaluable disease by RECIST 1.1 criteria. The ORR was 16.1% (80% CI: 11.5–21.9) for patients assigned docetaxel and apatorsen vs 10.9% (80% CI: 7.1–16.0) for those assigned docetaxel alone (one-sided *P* = 0.1531). Median duration of response was 6.2 months and 4.4 months in the docetaxel plus apatorsen and docetaxel alone responders, respectively.

Subgroup analysis was performed on patients with Bellmunt prognostic factors 0 vs 1–3 as well as those with time from prior systemic chemotherapy <3 vs ≥3 months. In participants with 0 risk factors, treatment with docetaxel and apatorsen vs docetaxel alone resulted in a median OS 14.3 vs 10.9 months (HR: 0.91, 80% CI: 0.59–1.42), compared to a median OS of 5.6 vs 5.1 months in those with 1–3 risk factors (HR: 0.77, 80% CI: 0.61–0.97). In participants with time from prior systemic chemotherapy ≥3 months, median OS was 8.0 vs 6.7 months for patients assigned docetaxel and apatorsen vs docetaxel alone (HR: 0.89, 80% CI: 0.67–1.18) and in those with time from prior systemic chemotherapy <3 months, the median OS was 5.9 vs 4.7 months (HR: 0.71, 80% CI: 0.53–0.96) for patients assigned docetaxel and apatorsen vs docetaxel, respectively.

There was also no significant impact on survival for patients having primary surgery, compared to those that did not (HR: 0.90; 80% CI: 0.73–1.12, one-sided *P* = 0.2638).

### Safety

Of the 200 participants randomised, 189 (93 assigned docetaxel plus apatorsen and 96 assigned docetaxel alone) initiated protocol treatment and were included in the safety population. Overall, 187 (98.9%) patients experienced an adverse event (AE) of any grade. The most common all-grade AEs were fatigue, anorexia, constipation, diarrhoea, nausea, anaemia, leukopaenia, and neutropaenia (Table 2). Among patients assigned to docetaxel and apatorsen, 77 (82.8%) had at least one grade 3–5 AE reported compared with 72 (75.0%) patients assigned to docetaxel alone. Common grade 3–5 AEs, including neutropaenia, leukopaenia, anaemia, and febrile neutropaenia, were well balanced between both groups. Patients treated with docetaxel and apatorsen had greater incidence of sepsis (15.1% vs 8.3%; *n* = 14 vs 8) and urinary tract infections (14% vs 7.3%; *n* = 13 vs 7) compared to those treated with docetaxel alone. Thirteen percent of patients discontinued treatment due to unacceptable adverse events (16.2% (*n* = 16) assigned docetaxel and apatorsen and 9.9% (*n* = 10) assigned docetaxel). Five patients in each arm experienced grade 5 AEs. In the docetaxel and apatorsen arm, one was possibly drug related to the treatment combination, and one was possibly drug related to apatorsen only. One death in each arm was possibly drug related to docetaxel alone.

**Table 2:** Selected adverse events among the safety population initiating assigned treatment

Treatment assignment								
	Patients given docetaxel and apatorsen (*n* = 93)				Patients given docetaxel (*n* = 96)			
	Grades 1–2	Grade 3	Grade 4	Grade 5	Grades 1–2	Grade 3	Grade 4	Grade 5
Fatigue	56 (60%)	7 (7%)	0	0	54 (56%)	12 (12%)	0	0
Diarrhoea	40 (43%)	7 (7%)	0	0	30 (31%)	5 (5%)	0	0
Anaemia	24 (26%)	16 (17%)	0	0	25 (26%)	10 (10%)	2 (2%)	0
Nausea	39 (42%)	2 (2%)	0	0	31 (32%)	3 (3%)	0	0
Anorexia	42 (45%)	0	0	0	29 (30%)	1 (1%)	0	0
Neutropaenia	4 (4%)	14 (15%)	19 (20%)	0	4 (4%)	11 (11%)	18 (19%)	0
Constipation	35 (38%)	2 (2%)	0	0	24 (25%)	1 (1%)	0	0
Dyspnea	22 (24%)	6 (6%)	0	0	26 (27%)	3 (3%)	1 (1%)	0
Leukopaenia	4 (4%)	18 (19%)	9 (10%)	0	4 (4%)	14 (14%)	5 (5%)	0
Alopecia	26 (28%)	0	0	0	26 (27%)	0	0	0
Peripheral neuropathy	27 (29%)	1 (1%)	0	0	22 (23%)	0	0	0
Creatinine increased	28 (30%)	2 (2%)	0	0	11 (11%)	1 (1%)	0	0
Muscle weakness	14 (15%)	4 (4%)	0	0	14 (14%)	4 (4%)	0	0
Oral mucositis	14 (15%)	1 (1%)	0	0	19 (20%)	2 (2%)	0	0
Vomiting	16 (17%)	4 (4%)	0	0	13 (13%)	3 (3%)	0	0
Dysgeusia	17 (18%)	0	0	0	17 (18%)	0	0	0
Hyponatremia	16 (17%)	7 (7%)	0	0	7 (7%)	4 (4%)	0	0
Urinary tract infection	7 (7%)	13 (14%)	0	0	6 (6%)	7 (7%)	0	0
Lymphopaenia	6 (6%)	11 (12%)	0	0	4 (4%)	6 (6%)	1 (1%)	0
Hypertension	7 (7%)	4 (4%)	0	0	5 (5%)	8 (8%)	0	0
Thrombocytopaenia	12 (13%)	0	0	0	10 (10%)	0	0	0
Sepsis	0	3 (3%)	9 (10%)	2 (2%)	0	1 (1%)	7 (7%)	0
Rash (maculopapular)	13 (14%)	0	0	0	8 (8%)	0	0	0
Febrile neutropaenia	0	9 (10%)	1 (1%)	0	2 (2%)	8 (8%)	0	0
Thromboembolic events	6 (6%)	3 (3%)	0	0	2 (2%)	5 (5%)	0	0
Intracranial haemorrhage	0	0	0	1 (1%)	0	0	0	1 (1%)
Cardiac arrest	0	0	0	0	0	0	0	1 (1%)
Colonic perforation	0	0	0	0	0	0	0	1 (1%)
Hepatic failure	0	0	0	0	0	0	0	1 (1%)
Death NOS	0	0	0	1 (1%)	0	0	0	1 (1%)
Multi-organ failure	0	0	0	1 (1%)	0	0	0	0

Selected grades 1–2 (in at least 10% of patients) and grades 3, 4, and 5 adverse events.

*NOS* not otherwise specified

### Exploratory analyses: serum Hsp27 levels

**Fig. 3:**
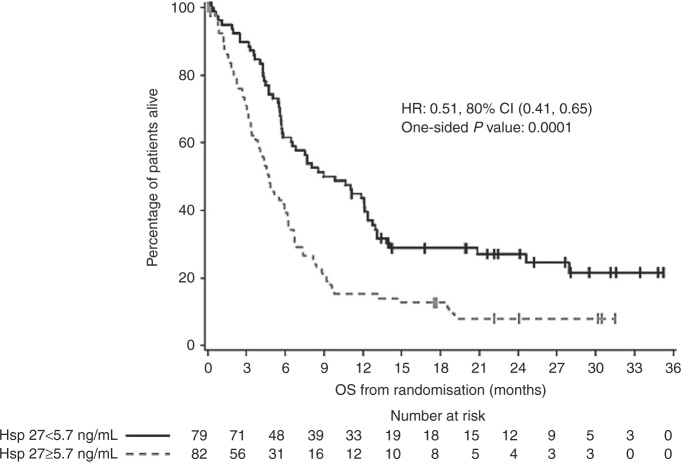
Kaplan–Meier estimate of overall survival (OS) according to baseline serum Hsp27 levels. One-hundred and sixty-one patients (80.5%) had baseline Hsp27 levels available. HR hazard ratio, CI confidence interval

Of the 200 participants, 161 (80.5%) had a baseline serum Hsp27 level available (80 in arm A and 81 in arm B). Median Hsp27 level was 5.7 ng/mL. In patients who had a baseline Hsp27 level <5.7 ng/mL (*n* = 79), median OS was significantly higher compared to those with a baseline level ≥5.7 ng/mL (*n* = 82) (median OS 9.4 vs. 4.7 months, HR: 0.51, 80% CI: 0.41–0.65, one-sided *P* = 0.0001, estimated 12-month OS 43.6% vs 15.2%) (Fig. 3). Treatment with docetaxel and apatorsen improved survival in both groups of patients with either baseline Hsp27 level <5.7 ng/mL (HR: 0.71, 80% CI: 0.50–1.00) or ≥5.7 ng/mL (HR: 0.67, 80% CI: 0.48–0.92; two-sided *P* = 0.87 for interaction) compared to docetaxel alone.

A landmark analysis was performed after cycle 2 to evaluate the change in Hsp27 level from baseline. Eighty patients (40 in each treatment group) had measurements available at both time points and median percentage change in serum Hsp27 from baseline to end of cycle 2 was an increase of 20.5% (range, −76.8 to +677.8%). In patients with a decrease or ≤20.5% increase of Hsp27, treatment with docetaxel and apatorsen significantly improved overall survival vs docetaxel alone (HR: 0.29, 80% CI: 0.18–0.48, median OS 12.2 vs 5.1 months) compared to those who had >20.5% increase in Hsp27 levels (HR: 0.77, 80% CI: 0.46–1.30, median OS 7.9 vs 6.8 months; two-sided *P* = 0.0727 for interaction).

## Discussion

The addition of apatorsen to docetaxel chemotherapy met its predefined end point resulting in improved overall survival compared to docetaxel alone in patients with metastatic urothelial carcinoma who are relapsed or refractory after a platinum-containing regimen. Although the improvement in median overall survival was 2 weeks, the hazard ratio and Kaplan–Meier curves show the overall benefit for the addition of apatorsen. However, these data are hypothesis generating and confirmatory trials are warranted to further study this targeted agent in patients with metastatic urothelial carcinoma.

Currently in the United States, single-agent immune checkpoint blockade with anti-PD-1/PD-L1 antibodies is the standard of care for patients following progression on platinum-based chemotherapy,^22–24^ and vinflunine is a cytotoxic approved for this indication in the European Union.^25^ While the development of immune checkpoint blockade has represented a breakthrough for patients with advanced urothelial carcinoma, the vast majority of patients fail to respond to single-agent PD-1 or PD-L1 inhibition with response rates of 15–25%.^22–24,26,27^ For these patients, there are no proven life-prolonging treatments, and the outlook remains quite bleak. Furthermore, efficacy results from phase II trials of many of these agents, which have led to accelerated FDA approval status, remain to be rigorously verified in the phase III setting and, in some cases, have not been confirmed in the subsequent phase trial.^28^ The phase III RANGE study, which evaluated ramucirumab plus docetaxel in platinum-refractory metastatic urothelial carcinoma, showed a modest PFS improvement of only 1.3 months compared to docetaxel plus placebo.^29^ Due to its gate-keeping statistical design, the study does not allow for formal testing of response rate unless OS benefit reaches HR 0.75 (currently immature). There were limited number of patients receiving immune checkpoint blockade prior and subgroup analysis did not show any benefit in patients with visceral metastasis. Therefore, novel agents that target chemotherapy-resistant urothelial carcinoma are still desperately needed.

Preclinical and smaller clinical data have shed light on the rationale for activity and benefit from ASO therapy targeting Hsp27. Hsp27 is a stress-activated, ATP-independent, cytoprotective chaperone that is upregulated in cancer and is associated with treatment resistance. Inhibition of Hsp27 expression in vitro and in vivo leads to increased sensitivity to cytotoxic chemotherapies.^14,15^ In addition, suppression of Hsp27 may lead to long-term dormancy in vivo in the absence of chemotherapy though inhibition of angiogenesis.^30^ Collectively, these findings may provide rationale for outcomes observed on this trial: while the median difference in overall survival was small, the hazard ratio suggests a 20% reduction in the risk of death over the course of the study associated with the combination treatment arm.

The biology of Hsp27 and its targeting by ASO has been shown to have challenges. Through the interplay of TNF-α and IL-10, Hsp27 is known to have anti-inflammatory effects.^31^ Further, while second-generation ASOs have modifications to allow greater nuclease resistance and increased binding affinities than their predecessors, potential toxicities include hybridisation-dependent toxicities—due to on- or off-target pharmacology—and hybridisation-independent toxicities due to nonantisense effects.^32^ Inhibition of Hsp27 by ASOs, therefore, may result in tumour suppression at the expense of increased inflammation. Indeed, in our study we noted a slightly higher incidence of sepsis (15.1% vs 8.3%; *n* = 14 vs 8) and urinary tract infections (14% vs 7.3%; *n* = 13 vs 7) in those treated with combination therapy. These safety signals are generally consistent with those seen in the Borealis-1 study, which evaluated platinum-based chemotherapy with or without apatorsen (600 or 1000 mg) vs chemotherapy plus placebo in the first-line setting.^20^ Toxicity was noted to be higher in the 1000 mg apatorsen arm compared to the 600 mg arm in that study, although the primary end point of improved OS was not met with either apatorsen dose compared to chemotherapy alone. In the phase III AFFINITY study of men with metastatic castration-resistant prostate cancer who progressed after docetaxel, custirsen, an ASO to clusterin, administered with chemotherapy showed no OS benefit vs chemotherapy alone.^33^ Our study, however, met its predefined end point and the future prospect of ASOs in urothelial carcinoma may depend on better patient selection, for example, with accurate biomarkers.

In our subgroup analysis, patients with lower baseline serum Hsp27 levels (<5.7 ng/mL) appeared to have better overall survival than those with higher baseline levels (≥5.7 ng/mL), irrespective of treatment. Therapy with apatorsen and docetaxel appeared to benefit both those with lower or higher Hsp27 levels, suggesting that baseline serum Hsp27 levels may act as a potential prognostic, but not predictive, biomarker in these patients. Furthermore, combination treatment with apatorsen and docetaxel appeared to benefit those with either a decline or limited increase of Hsp27 level ≤20.5% (HR: 0.29, 80% CI: 0.18–0.48) more so than those with an increase of >20.5% (HR: 0.77, 80% CI: 0.46–1.30) when comparing baseline and post cycle 2 levels. This finding may suggest that dynamic changes of Hsp27 levels in patients could serve as an indicator predicting benefit to combination treatment. Ultimately, these findings are simply hypothesis generating and may be informative for future trial designs.

These results should be interpreted in the context of study design. This was a randomised, controlled, comparative trial with one-sided 0.10 alpha-level test reflecting the objective to determine if the combination provided survival benefit relative to docetaxel alone. The rationale for this statistical design was that the addition of apatorsen to docetaxel was not felt to potentially yield a negative effect compared to docetaxel alone; however, this does allow for a higher false-positive rate and there is a potential for lead time bias with the apatorsen run-in. In the context of recent phase III results for immunotherapy agents in this space, our findings reinforce the importance of OS as a significant end point in well-designed later phase studies when evaluating potential practice-impacting treatments.^28^ To be eligible for our trial, patients must have received at least one prior platinum-based chemotherapy regimen, and no patient may have received more than two regimens for metastatic disease. While the current landscape is evolving for patients who are platinum-ineligible, and there are new options in the platinum-refractory setting (e.g., immunotherapy), this was not part of established treatment paradigm at the time of our study design. Therefore, the efficacy of apatorsen in patients having received immunotherapy is not currently assessed and granular data on subsequent lines of therapy are not available in this analysis. Furthermore, we only analysed serum Hsp27 levels at baseline and after cycle 2 as a potential marker of response to treatment. Evaluating the expression of Hsp27 measured by immunohistochemistry (IHC) in tumour tissue, as well as the effect of therapy on peripheral circulating tumour cells (CTCs), would be meaningful additional exploratory end points and, while not available in this current report, are planned future analyses.

In conclusion, the addition of apatorsen to docetaxel chemotherapy met its predefined survival end point in patients with refractory metastatic urothelial carcinoma in this phase II trial. These data are hypothesis generating and would require further study before informing clinical practice for this targeted therapy in metastatic urothelial carcinoma.

## Availability of data and material

All data and materials are available upon request.
